# A Review of the Evolution of Vision-Based Motion Analysis and the Integration of Advanced Computer Vision Methods Towards Developing a Markerless System

**DOI:** 10.1186/s40798-018-0139-y

**Published:** 2018-06-05

**Authors:** Steffi L. Colyer, Murray Evans, Darren P. Cosker, Aki I. T. Salo

**Affiliations:** 1 CAMERA—Centre for the Analysis of Motion, Entertainment Research and Applications, University of Bath, Bath, BA2 7AY UK; 2 Department for Health, University of Bath, Bath, BA2 7AY UK; 3 Department of Computer Science, University of Bath, Bath, BA2 7AY UK

**Keywords:** Automatic analysis, Body model, Cameras, Discriminative approaches, Gait, Generative algorithms, Motion capture, Rehabilitation, Sports biomechanics, Technique

## Abstract

**Background:**

The study of human movement within sports biomechanics and rehabilitation settings has made considerable progress over recent decades. However, developing a motion analysis system that collects accurate kinematic data in a timely, unobtrusive and externally valid manner remains an open challenge.

**Main body:**

This narrative review considers the evolution of methods for extracting kinematic information from images, observing how technology has progressed from laborious manual approaches to optoelectronic marker-based systems. The motion analysis systems which are currently most widely used in sports biomechanics and rehabilitation do not allow kinematic data to be collected automatically without the attachment of markers, controlled conditions and/or extensive processing times. These limitations can obstruct the routine use of motion capture in normal training or rehabilitation environments, and there is a clear desire for the development of automatic markerless systems. Such technology is emerging, often driven by the needs of the entertainment industry, and utilising many of the latest trends in computer vision and machine learning. However, the accuracy and practicality of these systems has yet to be fully scrutinised, meaning such markerless systems are not currently in widespread use within biomechanics.

**Conclusions:**

This review aims to introduce the key state-of-the-art in markerless motion capture research from computer vision that is likely to have a future impact in biomechanics, while considering the challenges with accuracy and robustness that are yet to be addressed.

## Key Points

1. Biomechanists aspire to have motion analysis tools that allow movement to be accurately measured automatically and unobtrusively in applied (e.g. everyday training) situations
2. Innovative markerless techniques developed primarily for entertainment purposes provide a potentially promising solution, with some systems capable of measuring sagittal plane angles to within 2°–3° during walking gait. However, accuracy requirements vary across different scenarios and the validity of markerless systems has yet to be fully established across different movements in varying environments
3. Further collaborative work between computer vision experts and biomechanists is required to develop such techniques further to meet the unique practical and accuracy requirements of motion analysis for sports and rehabilitation applications.

## Review

### Background

Vision-based motion analysis involves extracting information from sequential images in order to describe movement. It can be traced back to the late nineteenth century and the pioneering work of Eadweard Muybridge who first developed techniques to capture image sequences of equine gait [1]. Motion analysis has since evolved substantially in parallel with major technological advancements and the increasing demand for faster, more sophisticated techniques to capture movement in a wide range of settings ranging from clinical gait assessment [2] to video game animation [3]. Within sports biomechanics and rehabilitation applications, quantitative analysis of human body kinematics is a powerful tool that has been used to understand the performance determining aspects of technique [4], identify injury risk factors [5], and facilitate recovery from injury [6] or trauma [7].

Biomechanical tools have developed considerably from manual annotation of images to marker-based optical trackers, inertial sensor-based systems and markerless systems using sophisticated human body models, computer vision and machine learning algorithms. The aim of this review is to cover some of the history of the development and use of motion analysis methods within sports and biomechanics, highlighting the limitations of existing systems. The state-of-the-art technologies from computer vision and machine learning, which have started to emerge within the biomechanics community, are introduced. This review considers how these new technologies could revolutionise the fields of sports biomechanics and rehabilitation by broadening the applications of motion analysis to include everyday training or competition environments.

### General Principles and Requirements of Vision-Based Motion Analysis in Sports Biomechanics and Rehabilitation

Optical motion analysis requires the estimation of the position and orientation (pose) of an object across image sequences. Through the identification of common object features in successive images, displacement data can be “tracked” over time. However, accurate quantification of whole-body pose can be a difficult problem to solve since the human body is an extremely complex, highly articulated, self-occluding and only partially rigid entity [8–10]. To make this process more tractable, the structure of the human body is usually simplified as a series of rigid bodies connected by frictionless rotational joints.

Three-dimensional (3D) pose of rigid segments can be fully specified by six degrees of freedom (DOF): three relating to translation and three defining orientation. As such, even for a relatively simple 14-segment human body model, a large number of DOF (potentially as many as 84 depending on the anatomical constraints employed) need to be recovered to completely characterise 3D body configuration. From such a model, it is possible to compute joint angles, and with the incorporation of body segment inertia parameters, the whole body centre of mass location can be deduced, as in previous research in sprinting [11, 12], gymnastics [13] and rugby place-kicking [14]. Moreover, kinematic and kinetic data can be combined to allow the calculation of joint moments and powers through inverse dynamics analysis [15]. Such analyses have value across diverse areas from, for example, understanding lower-limb joint power production across fatiguing cycling efforts [16] to characterising joint torque profiles following anterior cruciate ligament reconstruction surgery [17]. However, obtaining accurate body pose is an essential step before reliable joint moments (and powers) can be robustly acquired, as inaccuracies in kinematic data will propagate to larger errors in joint kinetic parameters [18].

In certain cases within biomechanics, two-dimensional (2D) analyses using relatively simple body models suffice. Examples include when assessing movements which are considered to occur primarily in the sagittal plane such as walking [19] and sprinting [4], or when experimental control is limited, such as when ski jumpers’ body positions were analysed during Olympic competition [20]. Conversely, when the movement under analysis occurs in multiple planes, a multi-camera system and a more complex 3D model are required; for instance, when investigating shoulder injury risks associated with different volleyball spiking techniques [21]. The more extensive experimental set-ups for whole-body 3D analysis typically necessitate controlled laboratory environments and a challenge is to then ensure ecological validity (that movements accurately represent reality).

The main differences between 2D and 3D analysis relate to the complexity of the calibration and coordinate reconstruction processes, and joint angle definitions. Planar (2D) analysis can be conducted with only one camera, whereas at least two different perspectives are required to triangulate 2D information into 3D real-space coordinates [22, 23]. The number of DOF that need to be recovered (and consequently the number of markers required) in order to define segment (or any rigid body) pose differs between 2D and 3D methods. In 2D analysis, it is only possible to recover three DOF and this requires a minimum of two known points on the segment. Conversely, for 3D reconstruction of a rigid body, six DOF can be specified by identifying at least three non-collinear points.

A wide range of motion analysis systems allow movement to be captured in a variety of settings, which can broadly be categorised into direct (devices affixed to the body, e.g. accelerometry) and indirect (vision-based, e.g. video or optoelectronic) techniques. Direct methods allow kinematic information to be captured in diverse environments. For example, inertial sensors have been used as tools to provide insight into the execution of various movements (walking gait [24], discus [25], dressage [26] and swimming [27]). Sensor drift, which influences the accuracy of inertial sensor data, can be reduced during processing; however, this is yet to be fully resolved and capture periods remain limited [28]. Additionally, it has been recognised that motion analysis systems for biomechanical applications should fulfil the following criteria: they should be capable of collecting accurate kinematic information, ideally in a timely manner, without encumbering the performer or influencing their natural movement [29]. As such, indirect techniques can be distinguished as more appropriate in many settings compared with direct methods, as data are captured remotely from the participant imparting minimal interference to their movement. Indirect methods were also the only possible approach for biomechanical analyses previously conducted during sports competition [20, 30–33]. Over the past few decades, the indirect, vision-based methods available to biomechanists have dramatically progressed towards more accurate, automated systems. However, there is yet to be a tool developed which entirely satisfies the aforementioned important attributes of motion analysis systems.

### Historical Progression of Vision-Based Motion Analysis in Sports Biomechanics and Rehabilitation

#### Manual Digitisation

Manual digitisation was the most widespread motion measurement technique for many decades, and prior to digital technologies, cine film cameras were traditionally used [32, 34–36]. These were well-suited to the field of movement analysis due to their high image quality and high-speed frame rates (100 Hz in the aforementioned studies). However, the practicality of this method was limited by long processing times. With the advent of video cameras (initially tape-based before the transition to digital), cine cameras have become essentially redundant in the field of biomechanics. Digital video cameras are now relatively inexpensive, have increasingly high resolutions and fast frame rates (consumer cameras are generally capable of high-definition video at greater than 120 Hz, whereas industrial cameras are significantly faster), and are associated with shorter processing times.

Regardless of the technology used to capture motion, manual digitising requires the manual localisation of several points of interest (typically representing the underlying joint centres) in each sequential image from each camera perspective. Providing a calibration trial has been performed (where several control points of known relative location are digitised in each camera view), the position of the image body points can be reconstructed into real-space coordinates, most commonly via direct linear transformation [23]. Several software packages exist to aid this process and allow accurate localisation of points on rigid structures [37, 38]. Moreover, the repeatability of these methods has been supported by reliability analyses, for example within sprint hurdle [39] and cricket [33] research.

One of the primary advantages of manual digitising, which has allowed this method to persist as a means to collect kinematic data, is that the attachment of markers is not necessarily required. As such, manual digitisation remains a valuable tool particularly in sports biomechanics as it allows analysis of movement in normal training [12, 40, 41] and also competition [20, 30–33] environments without impeding the athlete(s). Additionally, this methodology provides a practical and affordable way of studying gait in applied therapy settings [42].

Unfortunately, a trade-off exists between accuracy and ecological validity when adopting field-based compared to laboratory-based optical motion analyses [43]. Specifically, the manual digitising approach can be implemented unobtrusively in applied settings with relative ease. However, the resultant 3D vector-based joint angles are difficult to relate to anatomically relevant axes of rotation. Moreover, if angles are projected onto 2D planes (in an attempt to separate the angle into component parts) movement in one plane can be incorrectly measured as movement in another, as discussed in relation to the assessment of elbow extension legality during cricket bowling [44]. Improvements to this early modelling approach are made by digitising external markers, such as medial and lateral condyles, which provide more accurate representations of 3D joint angles. However, certain drawbacks remain including the fact that manual digitising is a notoriously time-consuming and laborious task, and is liable to subjective error. These limitations have provided motivation for the development of automatic solutions made available by the emergence of more sophisticated technologies.

#### Automatic Marker-Based Systems

A large number of commercial automatic optoelectronic systems now exist for the study of human movement. The majority of these utilise multiple cameras that emit invisible infrared light, and passive markers that reflect this infrared back to the cameras and allow their 3D position to be deduced. Although the specifications of these systems differ markedly [45], the same underlying principles apply in the sense that several points of interest are located in sequential images, converted to real-space coordinates and used to infer 3D pose of the underlying skeleton. However, the primary difference between methodologies is that optoelectronic systems are capable of automatically locating large numbers of markers, substantially improving the time efficiency of this process. At least three non-collinear markers must be affixed to each segment to specify six DOF. If only two joint markers are used to define a segment, the same challenges exist as those outlined above in relation to manual digitisation. Increasing the number of markers attached to each segment increases the system’s redundancy. However, extensive marker sets encumber the natural movement pattern and tracking marker trajectories can become challenging if markers are clustered close to one another or become occluded [45].

The accuracies of several widely utilised commercial marker-based systems have been evaluated using a rigid, rotating structure with markers attached at known locations [45]. Root mean square errors were found to be typically less than 2.0 mm for fully visible moving markers and 1.0 mm for a stationary marker (errors were scaled to a standard 3-m long volume) indicating excellent precision when markers are attached to a rigid body. However, the exact placement of markers on anatomical landmarks is difficult to realise and markers placed on the skin do not directly correspond to 3D joint positions. Various protocols exist to locate joint centres and/or define segment pose from markers placed on anatomical landmarks; however, these different conventions produce varying out-of-sagittal plane results when compared over the same gait cycles [46]. In fact, there is also inevitable day-to-day and inter-tester variability in marker placement, which reduces the reliability of marker-based measurements, particularly for transverse plane movements [47, 48].

It is well acknowledged that the rigid body assumption (underlying marker-based motion analysis) can be violated by soft tissue movement, particularly during dynamic activities [49]. This phenomenon has been consistently demonstrated by studies which have compared marker-derived kinematics with those using “gold standard” methods such as fluoroscopy [50], Roentgen photogrammetric techniques [51, 52] and intra-cortical bone pins [53–55]. As soft tissue movement introduces both systematic and random errors of similar frequency to the actual bone movement, this is difficult to attenuate through data smoothing [56]. Over the last two decades, careful design of marker sets [57] and the use of anatomical calibration procedures [58] have somewhat alleviated this measurement artefact. For example, initial static calibration trials can be captured whereby joint centres and segment coordinate systems are defined relative to markers. It is then possible to remove certain markers that are liable to movement during dynamic movement, without compromising the deduction of segment pose [59–61]. Moreover, the placement of marker clusters (typically three or four non-collinear markers rigidly affixed to a plate) not only provides a practical method to define the segment’s six DOF, but can also be strategically positioned to reduce soft tissue artefact [49]. The development of more sophisticated pose-estimation algorithms [62] and joint angle definitions [43] has further advanced the accuracy of marker-based analyses.

Optoelectronic systems are also relatively sensitive to the capture environment. In particular, sunlight, which includes a strong infrared component, can introduce undesirable noise into the measurements. In the past, marker-based analysis has therefore been restricted to indoor conditions (where light conditions could be stringently controlled). However, innovative active filtering features can alleviate these errors and have even allowed data to be captured during outdoor snow sports [63].

It is, therefore, clear that optoelectronic systems have made significant advancements in recent times within the field of biomechanics. However, even though careful methodological considerations can improve the accuracy of the data acquired, several limitations remain, including long participant preparation times, potential for erroneous marker placement or movement, and the unfeasibility of attaching markers in certain settings (e.g. sports competition). Perhaps one of the most fundamental problems is the physical and/or psychological constraints that attached markers impart on the participant, influencing movement execution. These drawbacks can limit the utility of marker-based systems within certain areas of sports biomechanics and rehabilitation, and have driven the exploration of potential markerless solutions.

### Markerless Motion Analysis Systems

An attractive future advancement in motion analysis is towards a fully automatic, non-invasive, markerless approach, which would ultimately provide a major breakthrough for research and practice within sports biomechanics and rehabilitation. For example, motion could be analysed during normal training environments more readily, without the long subject preparation times associated with marker-based systems or the laborious processing required for manual methods. Moreover, it could provide a potential solution for a common dilemma faced by biomechanists, which stems from the trade-off between accuracy (laboratory-based analyses) and external validity (field-based analyses).

Markerless methods are not yet in widespread use within biomechanics, with only a small number of companies providing commercial systems (details for a selection of which are provided in Table 1). However, it remains unclear exactly what precision these systems can achieve in comparison to the other, more established motion analysis systems available on the market. Certainly, the technology is under rapid development with modern computer vision algorithms improving the robustness, flexibility and accuracy of markerless systems. A number of reviews [10, 64–67] have been previously published detailing these developments, targeting specific application areas such as security, forensics and entertainment. The aim of this section is to introduce the biomechanics community to the current state-of-the-art markerless technology from the field of computer vision, and to discuss where the technology stands in terms of accuracy.

**Table 1:** A selection of commercially available full-body markerless systems

Company	Cameras	Capture environments	Integration with other biomechanical tools	Real-time capacity
Captury Studio Ultimate The Captury www.thecaptury.com	Unlimited number with combination of resolutions	No specific background necessary. Can handle dynamic scenes and illumination changes, as long as sufficient contrast.	None. Applications primarily within entertainment	Yes
BioStage Organic Motion www.organicmotion.com	8–18 (120 fps in real-time)	Laboratory-based	Force plates and electromyography	Yes
Shape 3D Simi www.simi.com	Up to 8 high-speed colour cameras	Can operate outdoors but stable background with good contrast is required	Force plates, electromyography and pressure sensors	No

Information obtained from company web-pages (accessed July 2017)

#### Recent Computer Vision Approaches to Markerless Motion Capture

In marker-based motion capture, cameras and lighting are specially configured to make observation and tracking of markers simple. Where multiple markers are used, individual markers need to be identified, and measurements then taken either directly from the positions of the markers, or from inferring the configuration of a skeleton model that best fits the marker positions. Markerless systems have some similarity to this, with differences mostly induced by the significantly more difficult process of gathering information from the images.

**Fig. 1:**
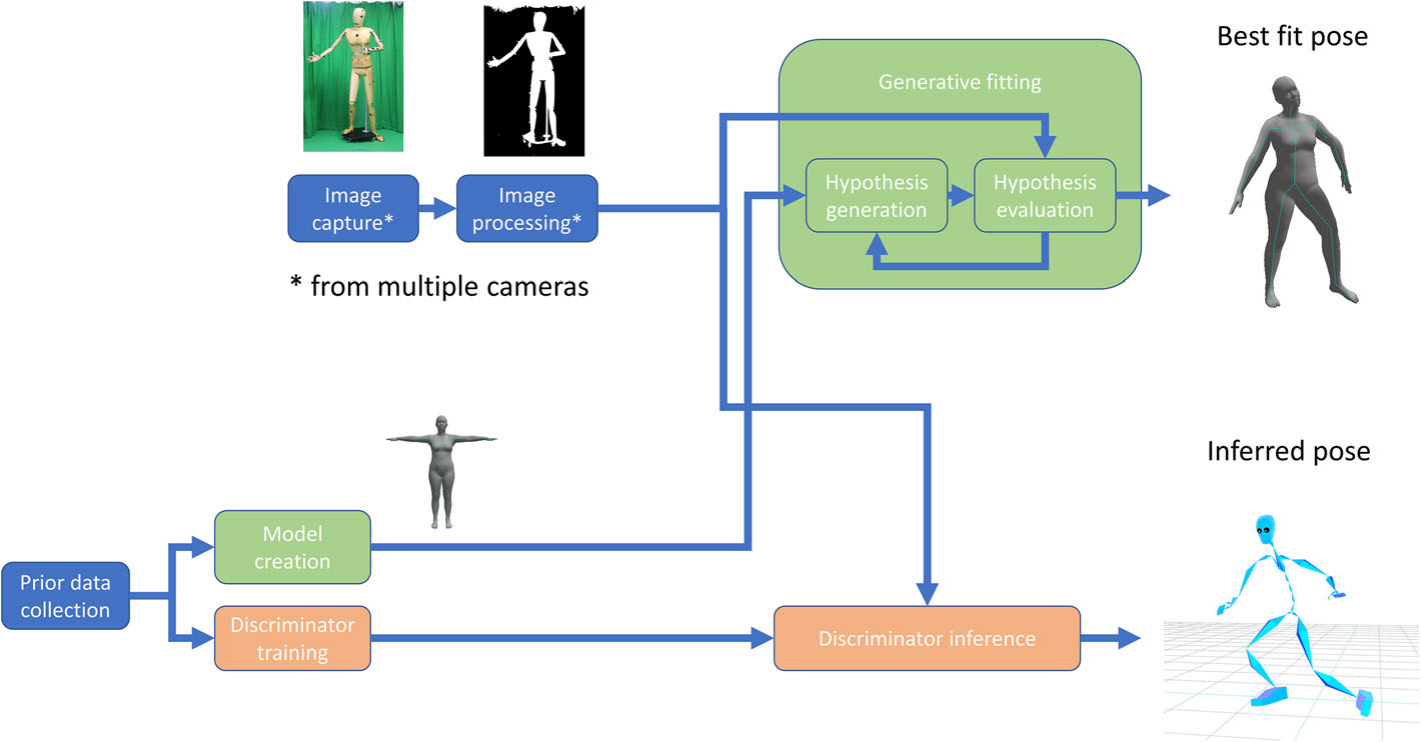
General structure of a markerless motion capture whether using generative (green) or discriminative (orange) algorithms

The four major components of a markerless motion capture system are (1) the camera systems that are used, (2) the representation of the human body (the body model), (3) the image features used and (4) the algorithms used to determine the parameters (shape, pose, location) of the body model. The algorithms used to infer body pose given image data are usually categorised as either “generative” (in which model parameters can be used to “generate” a hypothesis that is evaluated against image data and then iteratively refined to determine a best possible fit) or “discriminative” (where image data is used to directly infer model parameters). In general, a markerless motion capture system will have the form shown in Fig. 1. This consists of an offline stage where prior data inform model design or training of a machine learning-based discriminative algorithm, and then image data are captured, processed and input into the algorithms that will estimate body pose and shape.

#### Camera Systems for Markerless Motion Capture

**Fig. 2:**
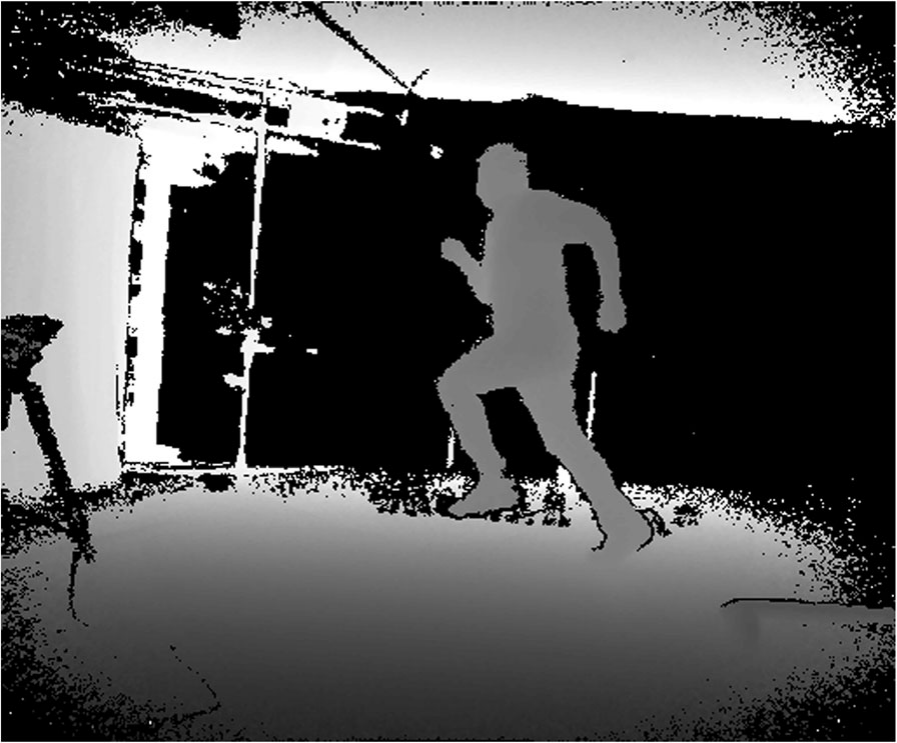
Example of a depth map. Brighter pixels are further away from the camera. Black pixels are either too far away or on objects that do not reflect near infrared light

Two major families of camera systems are used for markerless motion capture differing by whether or not a “depth map” is produced. A depth map is an image where each pixel, instead of describing colour or brightness, describes the distance of a point in space from the camera (Fig. 2). Depth-sensing camera systems range from narrow-baseline binocular-stereo camera systems (such as the PointGrey Bumblebee or the Stereolabs Zed camera) to “active” cameras which sense depth through the projection of light into the observed scene such as Microsoft’s Kinect. Depth information can help alleviate problems that affect traditional camera systems such as shadows, imperfect lighting conditions, reflections and cluttered backgrounds. Active, depth-sensing camera systems (often termed RGB-D cameras where they capture both colour and depth) have proven effective for real-time full-body pose estimation in interactive systems and games [68, 69]. The devices most commonly use one of two technologies: structured light or time-of-flight (ToF). Structured light devices sense depth through the deformations of a known pattern projected onto the scene, while ToF devices measure the time for a pulse of light to return to the camera. The two technologies have different noise characteristics and trade-offs between depth accuracy and spatial resolution [70]. The most well-known active cameras are Microsoft’s original structured light “Kinect”, and the replacement ToF based “Kinect For Xbox One” (often referred to as the Kinect 2), which are provided with body tracking software designed for interactive systems. The performance of this tracking system has been analysed for both versions of the cameras [71], but clearly falls well below the accuracy required for precision biomechanics (it could be speculated, however, that a tracking system not dedicated to interactive systems may achieve greater accuracy using these devices). Active cameras have been applied to sports biomechanics [72, 73] using bespoke software, but current hardware limitations (effective only over short range, maximum 30 Hz framerates, inoperability in bright sun light, and interference between multiple sensors) are likely to limit their application in sports biomechanics for the foreseeable future.

#### Body Models

**Fig. 3:**
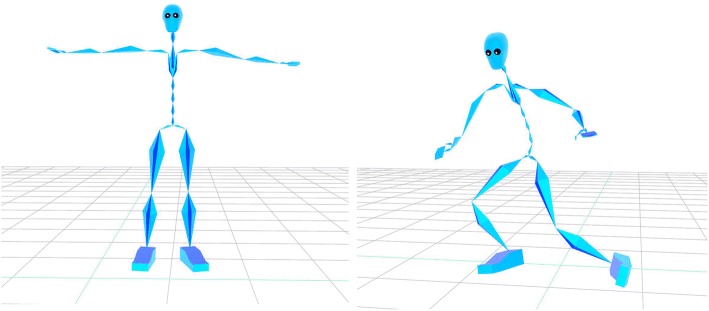
Example of a poseable skeleton model. “Bones” of a pre-specified length are connected at joints, and rotation of the bones around these joints allows the skeleton to be posed. The skeleton model is commonly fit to both marker-based motion capture data and computer vision-based markerless systems

The body models used by markerless motion capture are generally similar to those used by traditional marker-based approaches. A skeleton is defined as a set of joints and the bones between these joints (Fig. 3). The skeleton is parameterised on the lengths of the bones and the rotation of each joint with pose being described by the joint angles. For discriminative approaches, this skeleton model can be enough, but generative approaches will also require a representation of the person’s volume.

**Fig. 4:**
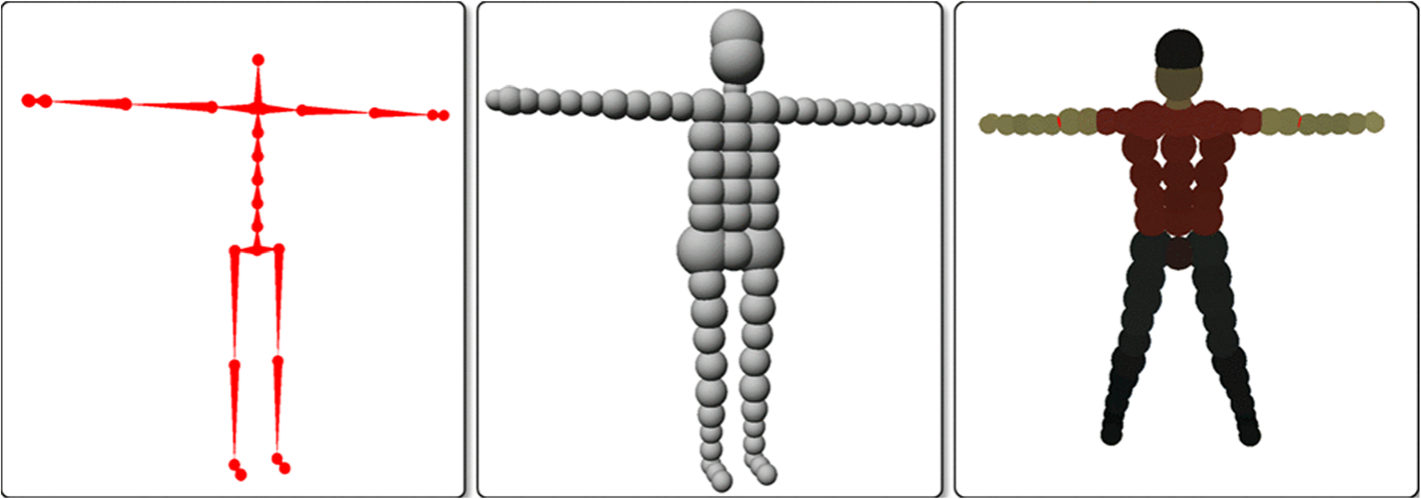
Sum of Gaussian body model from Stoll [75]. A skeleton (left) forms the foundation of the model, providing limb-lengths and body pose. The body is given volume and appearance information through the use of 3D Spatial Gaussians arranged along the skeleton (represented by spheres). The resulting information allows the model to be fit to image data

In earlier works, the volume of the model is represented by simple geometric shapes [74] such as cylinders. Such models remain the state-of-the-art within computer vision in the form of a set of “spatial 3D Gaussians” [75] attached to the bones of a kinematic skeleton (Fig. 4). The advantage of this representation has been to enable fast, almost real-time fitting in a generative framework using passive cameras and a very simple set of image features.

**Fig. 5:**
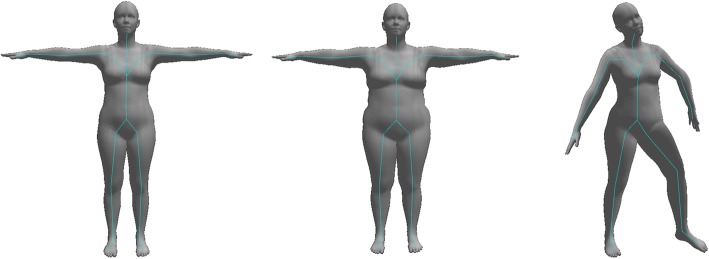
Skinned Multi-Person Linear Model (SMPL) [79] body model. This model does not have an explicit skeleton. Instead, the surface of a person is represented by a mesh of triangles. A set of parameters (learnt through regression) allows the shape of the model to be changed from a neutral mean (left) to a fatter (middle) or thinner, taller, or other body shape. Once shaped, the centres of joints are inferred from the neutrally posed mesh, and then the mesh can be rotated around these joints to produce a posed body (right)

In general though, the trend has been to use 3D triangle meshes common in graphics and computer games, either created by artists, as the product of high-definition 3D scans [76], or more recently, by specialising a generic statistical 3D shape model [77–79] (Fig. 5). Statistical body shape models allow a wide range of human body shapes to be represented in a relatively small number of parameters and improve how the body surface deforms under joint rotations. However, because these models focus on the external surface appearance of the model, the underlying skeleton is questionably representative of an actual human skeleton and as such, care must be taken should these models be used for biomechanical measurements.

The parameterisation of the human body used by motion capture models is always a simplification, and although capable of a realistic appearance, can also represent physically unrealistic shapes and poses. If the algorithms are not carefully constrained, these solutions can appear optimal for the available data. To ensure only physically realistic solutions are produced, the algorithms must be supported by constraints on the body model such as explicit joint limits [80] or probabilistic spaces of human pose and motion deduced by machine learning [81]. In either case, there exists a balance between enforcing the constraints and trusting the observed data to achieve a solution that is both plausible and precise.

#### Image Features for Markerless Motion Capture

A digital image, fundamentally, is a 2D grid of numbers each representing the brightness and colour of a small region, or pixel. The process of determining how pixels relate to objects is a fundamental task in computer vision, and there have been many proposed approaches to extracting “features” from an image that are meaningful. It is the great difficulty of this task that marker-based systems have been developed to avoid.

**Fig. 6:**
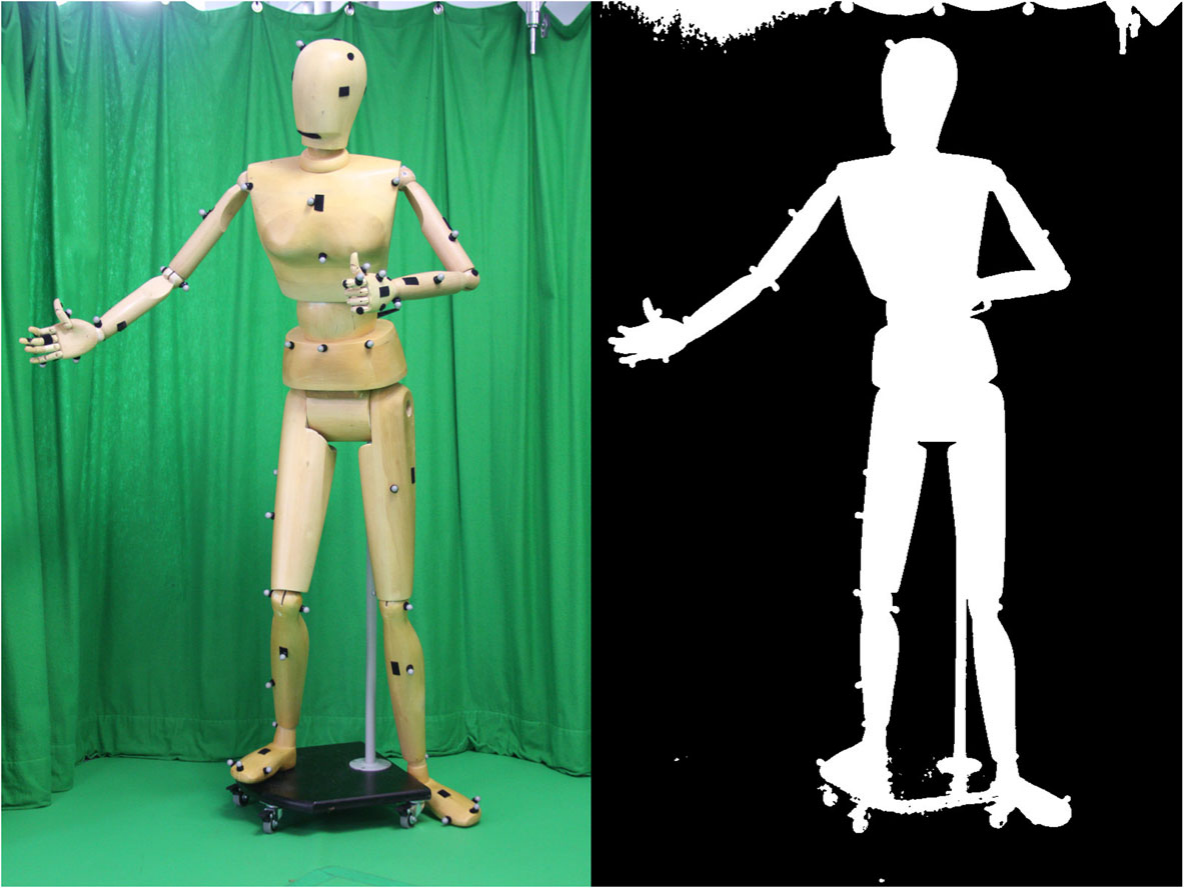
Silhouette on the right from chroma keying the image on the left. When seen as only a silhouette, it is not possible to infer if the mannequin is facing towards or away from the camera

For motion capture, the primary aim is to determine the location and extents in the image of the person being captured. The earliest and one of the most robust approaches to this task is termed chroma keying*.* This is where the background of the scene is painted a single specific colour, allowing the silhouette of the person (who is dressed in a suitable contrasting colour) to be easily segmented (Fig. 6). For environments where chroma keying is not possible, there are a large number [82] of background subtraction algorithms. However, these can all suffer from problems with shadows, lighting changes, reflections and non-salient motions of the background (such as a crowd or other athletes).

**Fig. 7:**
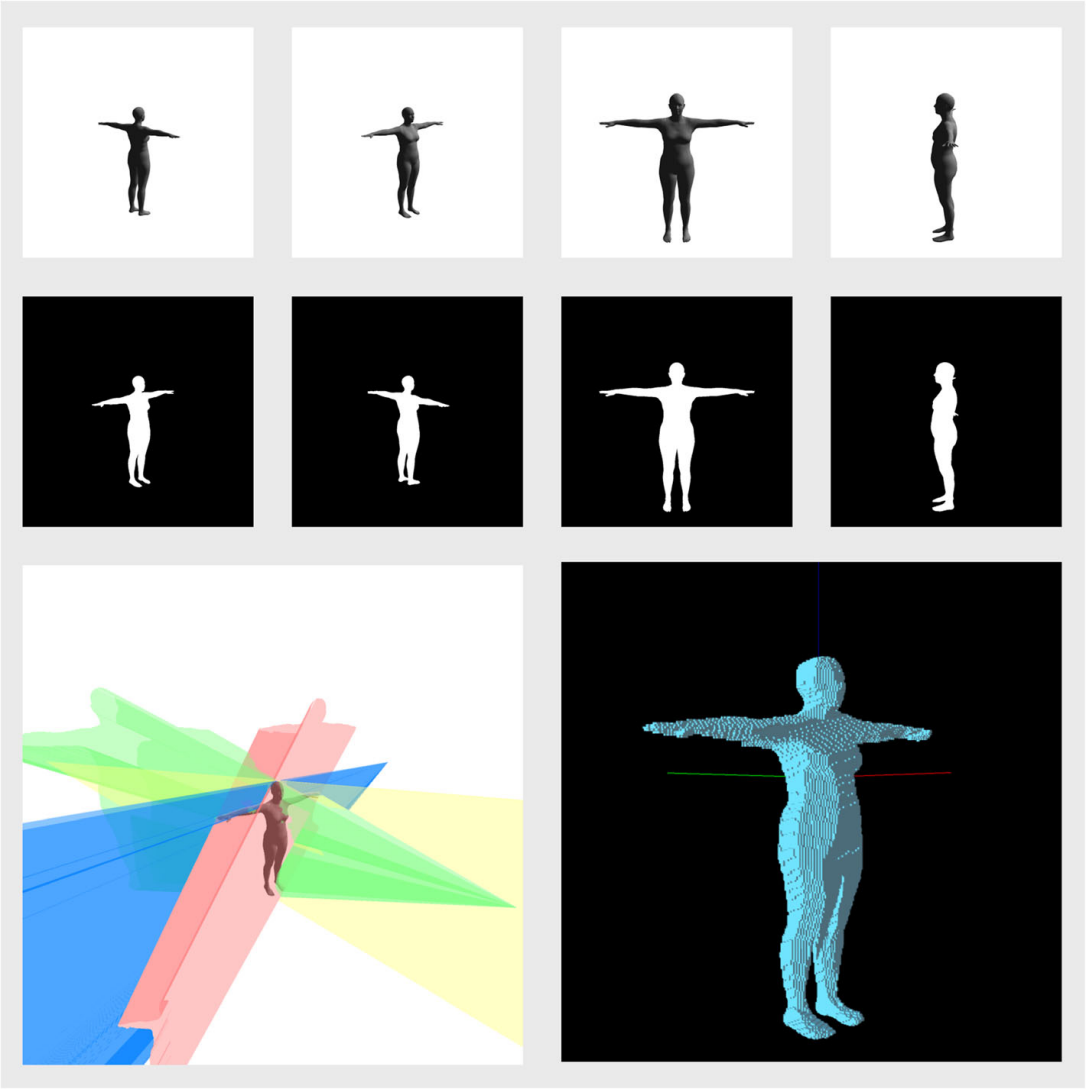
The generation of a visual hull, which is a type of 3D reconstruction of an object viewed from multiple cameras. Top row: images of an object are captured as 2D images from multiple directions. Middle row: these images are processed to produce silhouette images for each camera. Bottom left: the silhouettes are back-projected from each camera, resulting in cone-like regions of space. Bottom right: the intersection of these cones results in the visual hull

Image silhouettes are also inherently ambiguous and provide no information on whether the observed subject is facing towards or away from the camera (Fig. 6). This ambiguity can only be reduced by the use of extra cameras or more sophisticated image features. Where large numbers of cameras are available, silhouettes can be combined into a 3D representation known as a visual hull [83], which is an approximation of the space occupied by the observed person (Fig. 7). More sophisticated 3D reconstructions can also be carried out [84]; however, any added accuracy must be traded off against increased computational complexity. Improving the reconstruction does not fully resolve all fitting difficulties, however, and extra information that identifies which regions of the silhouettes correspond to which regions of the body are often needed to completely resolve all possible confusion [85]. Nevertheless, silhouettes have formed a key aspect of many markerless motion capture works including the work of Corazza et al. [86], which has reported some of the most accurate results for automatic markerless body motion capture, and Liu et al. [87], which enables the kinematic motion analysis of multiple persons. The trend, however, has been to move away from the use of image silhouettes to improve robustness, reduce ambiguities, reduce the number of cameras and simplify capture procedures. In this regard, the work of Stoll et al. [75] is significant for enabling the body model to fit to the image using only a simple colour model, while the advent of deep learning [88] and its provision of robust and fast body-part detectors has made dramatic improvements on what can be done outside of laboratory conditions [89, 90], including recognising body pose of many people from a single uncalibrated and moving camera [91].

#### Generative Algorithms

In generative motion capture approaches, the pose and shape of the person is determined by fitting the body model to information extracted from the image. For a given set of model parameters (body shape, bone lengths, joint angles), a representation of the model is generated. This representation can then be compared against the features extracted from the image and a single “error value” calculated, which represents how much the hypothesis differs from the observed data. In one possibility, the 3D triangle mesh resulting from the predicted parameters can be projected into the 2D image, and the overlap of the mesh and the silhouette of the person can be maximised [92]. Alternatively, the 3D body model can be compared against a 3D reconstruction such as a visual hull by minimising the distances between the 3D vertices of the model, and the 3D points of the visual hull [86, 93] through a standard algorithm known as iterative closest point.

A key factor of generative approaches is the appropriate definition of the function that compares a specific hypothesis with the information available in the images. If this is not carefully considered, then the search for the optimal set of model parameters can easily fail, resulting in poor estimates or nonsense configurations where joints bend at unrealistic angles and limbs penetrate inside the body. Constructing a cost function that is robust to image noise and to unrealistic model configurations is difficult meaning generative models often need a reliable initial guess of the model parameters. In extremes this would mean forcing the person being captured to assume a specific pose at the start of tracking. If the fitting then becomes confused by occlusions, image noise or other failure, tracking will not be able to correct itself without manual intervention. Researchers have attempted to address this situation using improved searching algorithms [92], extra information derived from robust body part detectors [90] and recent pose-recognition algorithms [94–97], or by coupling generative methods with discriminative methods [98].

#### Discriminative Approaches

Discriminative algorithms avoid the process of iteratively tuning the parameters of a body model to fit the image and as such they are also often referred to as model-free algorithms. Compared with generative approaches, they will often have a much faster processing time, improved robustness and reduced dependence on an initial guess. However, they can have reduced precision, and they require a very large database of exemplar data (far more than is required even for constructing the statistical body shape models used by generative algorithms) from which they can learn how to infer a result.

Discriminative approaches have two major families. One approach is to discover a mapping directly from image features to a description of pose, such as by using machine learning-based regression [99, 100]. In this way, it is possible to “teach” the computer how to determine the pose of a simple skeleton model using only the image data. The most recent approaches in this family use deep learning to train a system that can identify the body parts of multiple people, the probable ownership of joints, and then quickly parse this to determine skeletons [91]. Alternatively, a database of pose examples can be created and then searched to discover the most similar known pose given the current image, as used in previous studies [101–103].

The main difficulty in the use of discriminative algorithms is the creation of the exemplar data. If the available data is insufficient, then poses, physiques and even camera positions that were not suitably represented will lead to false results as the system will not be able to generalise from what it “knows” to what it “sees”. This will also affect the precision of the result because the algorithm is restricted to giving solutions close to what it “knows” about, so small variations may not be fully represented in the results. As a result, discriminative approaches are used as initial guesses for generative approaches [98].

#### Summary of Markerless Approaches

The current state-of-the-art shows the computer vision community aiming to develop solutions to markerless motion capture that are applicable and reliable outside of laboratory conditions. Although carefully calibrated silhouette-based algorithms using sophisticated subject-specific body models have shown the most accurate results to date, they have been limited to laboratory conditions using a large number of cameras [86]. By taking advantage of modern technologies such as improved solvers [92], advanced image features and modern machine learning [100], recent works are providing solutions that reduce the required number of cameras [104], allow moving cameras [105], increase the number of people that can be tracked and provide robust detection and fitting in varied environments [91]. The ability to do this without knowledge of camera calibration further improves the potential ease of use of future systems; however, calibration is likely to remain a necessity where precise measurement is needed such as in biomechanics.

#### Accuracy of Current Markerless Motion Capture Systems

There are distinct differences in the accuracy requirements between motion analysis techniques in the fields of computer vision and biomechanics, which must be taken into account when attempting to apply computer vision methods more broadly across other disciplines. For instance, accuracy within computer vision (primarily entertainment applications) is typically assessed qualitatively and is primarily evaluated based on appearance. Conversely, in biomechanical settings, it is fundamental that any motion analysis system is capable of robustly quantifying subtle differences in motion, which could be meaningful from a musculoskeletal performance or pathology perspective. Nonetheless, there is no general consensus regarding the minimum accuracy requirement of motion analysis systems and the magnitudes of the inevitable measurement errors will vary depending on the context (laboratory vs. field), the characteristics of the movement and the participant, the experimental setup and how the human body is modelled.

As previously described, marker-based approaches are currently the most widely used systems in biomechanical laboratories. However, a prominent source of measurement error in marker-based systems is skin movement artefact [56], which violates the rigid body assumption underlying these methods. Reports suggest that errors due to soft tissue movement can exceed 10 mm for some anatomical landmarks and 10° for some joint angles when compared to more precise, yet invasive, methods (e.g. intra-cortical bone pins) [106]. However, these errors in joint angle measurements may be reduced to 2°–4° by using more sophisticated pose-estimation algorithms such as global optimisation (inverse kinematics) with joint constraints [62]. As the “gold standard” methods are inappropriate in many contexts, and marker-based systems are the most frequently utilised motion analysis technique in the field, agreement between markerless and optoelectronic systems would be considered to provide evidence for the validity of markerless motion analysis techniques.

There are already studies which have attempted to evaluate the accuracy of markerless systems (summarised in Table 2) by comparing the kinematic output variables against those obtained using marker-based optoelectronic systems [10, 86, 93, 107–109] or manual digitisation [110]. These validations mostly study relatively slow movements (typically walking gait), whereas to verify the utility of these approaches in sports applications, much quicker movements need to be thoroughly assessed. One clear observation from these results is that transverse plane rotations are currently difficult to extract accurately and reliably by markerless technologies [107, 110].

**Table 2:** Overview of studies comparing markerless with conventional motion analysis systems

Publication	Movement(s) analysed	Markerless system description	Procedure/system for comparison	Number of cameras	Outcome
Trewartha et al. [110]	Starjump, somersaults	Gen-locked video cameras (50 Hz), subject-specific model	Manual digitising (TARGET system)	3	RMS differences for three movements ranged from 10 mm and 30 mm for pelvis location and between 2° and 8° for body configuration angles.
Corazza et al. [93]	Walking	Visual hull construction and a priori subject-specific model	Virtual environment (Poser software)	16	RMS errors of hip, knee and ankle angles ranged from 2.0° (hip abduction/adduction) to 9.0 (ankle dorsi/plantar flexion)
Mündermann et al. [10]	Walking	Video cameras (75 Hz), visual hull construction and a priori subject-specific model	Qualisys (120 Hz)	8	Average knee joint angle deviation: 2.3° (sagittal plane) and 1.6° (frontal plane).
Corazza et al. [86]	Walking	Video cameras (120 Hz), visual hull construction and a priori subject-specific model	Qualisys (120 Hz)	8	Average deviations between joint (hip, knee, ankle, shoulder, elbow and wrist) centres: 15 mm mean absolute error (ranged from 9 to 19 mm)
Choppin and Wheat [72]	Reaching, throwing, jumping	Microsoft Kinect (30 Hz)	Motion Analysis Corporation (60 Hz)	1 Kinect, 12 optoelectronic	Flexion/extension and abduction/adduction of hip, knee, elbow and shoulder; shoulder plane and elevation studied. Maximum abduction error: 44.1° and 13.9°. Maximum flexion error: 36.2° and 19.5° (NITE and IPIsoft tracking algorithms, respectively)
Ceseracciu et al. [107]	Walking	BTS SMART-D (100 Hz)	BTS SMART-D (200 Hz)	8	Maximum RMS differences range: 11.0° (ankle dorsi/ plantar flexion) to 34.7° (hip internal/external rotation)
Sandau et al. [108]	Walking	Monochrome cameras (75 Hz), unconstrained articulated model fit to 3D point clouds (aided by full body patterned suit)	Ariel Performance Analysis System	8	RMS differences in lower limb 3D angles ranged between 1.8° (hip abduction/adduction) and 4.9° (hip internal/external rotation)
Ong et al. [109]	Walking and jogging	Point Grey cameras (25 Hz)	Motion Analysis Corporation (100 Hz)	2 markerless, 8 marker-based	RMS differences ranged from 0.2° (knee abduction/adduction of jogging) to 1.0° (ankle dorsi/plantar flexion of walking). Significant differences between markerless and marker-based for the ankle joint angles.

*RMS* root mean square

**Fig. 8:**
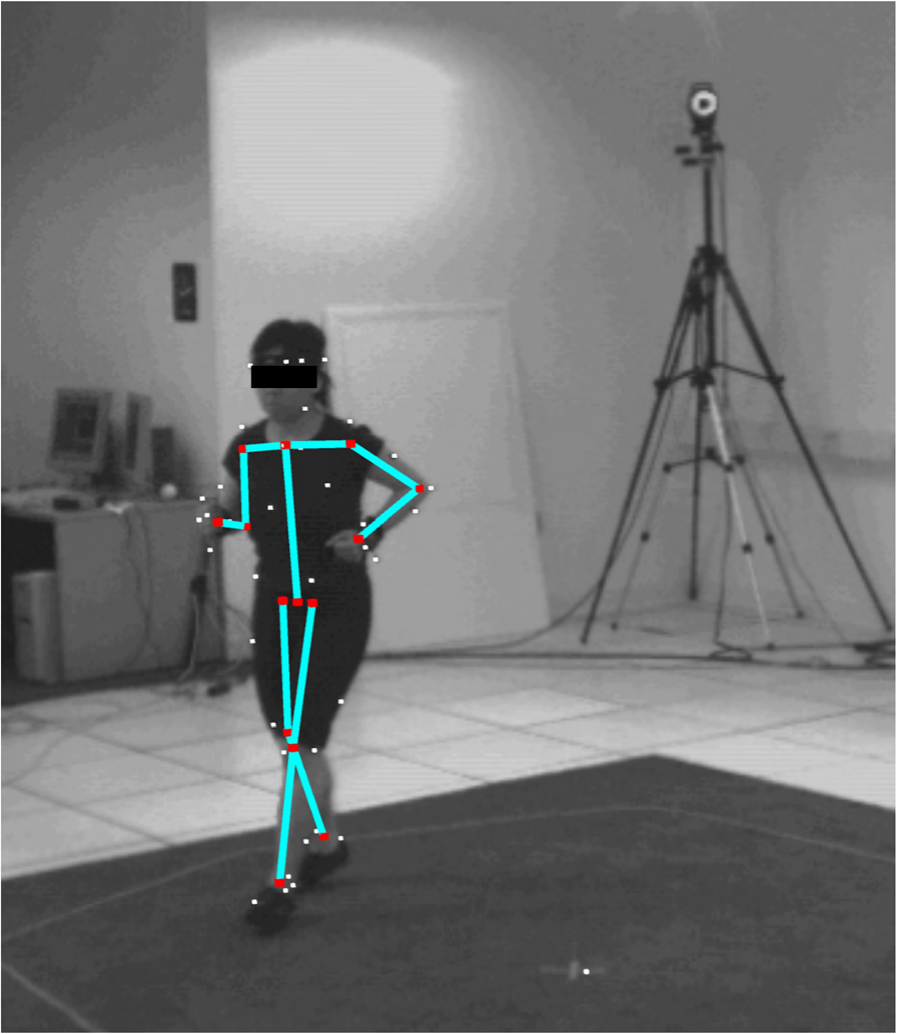
An example image from the HumanEva dataset used to validate markerless systems within computer vision. White dots indicate the location of tracked reflective markers and the cyan lines represent the defined skeleton model fit to the marker data. Although useful as an early benchmark for markerless tracking systems, the dataset has clear limitations for assessing the quality of any markerless tracking results, especially in the context of biomechanics. Notice that the markers are attached to clothing, marker clusters are not utilised, and the joint centres inferred from the fitted skeleton are not closely aligned with how the person appears in the image (e.g. right elbow and hip joints). See further information in the text

In the computer vision community, it is common practice to advance technology by establishing benchmark datasets against which many authors can rank their algorithm’s performance. Two such benchmarks are the widely used HumanEva dataset [111] and the more recent Human 3.6M dataset [112]. These datasets provide video of people performing actions (walking, jogging, boxing etc.) while also being tracked with marker-based tracking systems. Table 3 shows a sample of published comparison results for the HumanEva dataset. These results show that precision of markerless techniques remains too low to be applicable for biomechanics analyses. However, the video data and motion capture data in the HumanEva dataset are themselves of limited quality. For example, videos are low resolution and camera placement is sub-optimal, while markers are limited and sub-optimally located (often on relatively loose clothing) with no marker clusters to aid tracking (Fig. 8). For comparison, the results of Corazza et al. [86] on HumanEva have a mean joint centre position error of 79 ± 12 mm, while on the authors’ own higher resolution data with better camera and marker placement, a much smaller 15 ± 10 mm error was achieved.

**Table 3:** Selection of published validation results against the HumanEva datasets

Publication	3D joint position error (mm)	Standard deviation of error (mm)
Corazza et al. [86]	79.0	11.5
Amin et al. [94]	54.5	
Belagiannis et al. [95]	68.3	
Saini et al. [92]	45.7	5.3
Guo et al. [103]	46.8	
Elhayek et al. [90]	66.5	
Rhodin et al. [98]	54.6	24.2
Bogo et al. [89]	79.9

The discrepancies observed by Corazza et al. [86] between the validation results against the HumanEva benchmark and the more rigorously captured marker-based data show the difficulties of treating marker-based motion capture as the criterion method. In fact, although these benchmarks are useful for showing the general performance of different algorithms, neither the marker-based nor manual digitising methods used to validate markerless technologies can provide exact “true” body pose due to experimental artefacts that are inevitably introduced. Additionally, ensuring a close match between the body model applied to both systems is a challenge, which may necessitate “off-line” stages perhaps involving imaging, as in previous work [76]. Adding markers to the validation images might also unduly bias the performance of a markerless system under test, if the algorithm detects the markers and uses them for its benefit or if the markers adversely affect the performance of the markerless algorithm (altering silhouette shapes, for example) [107]. As such, alternative methods of validating the performance of markerless systems have also been considered, such as utilising force plate data to analyse centre of mass movement [113] and creating virtual environments (synthesised images) in which a predefined model moves with known kinematics [93]. Although synthesised images can be invaluable for developing an algorithm (synthetic images were used for generating training images for Microsoft’s Kinect pose tracker [68]), the idealised image data is unlikely to capture the noise and error sources of real imagery.

### Future of Markerless Approaches to Analyse Motion in Sports Biomechanics and Rehabilitation

It is clear that a broad range of markerless technologies have emerged from computer vision research over recent times, which have the potential to be applied across diverse disciplines and settings. The priorities and requirements for a markerless motion capture system will depend on the research area and the unique capture environment, and are thus non-uniform across disciplines. In sports biomechanics and rehabilitation applications, motion analysis systems must be highly accurate in order to detect subtle changes in motion, as well as being adaptable, non-invasive and unencumbering. With these system requirements in mind, the current progression of technologies suggests that the future of practical markerless motion capture will lie with techniques such as those presented by Elhayek et al. [90], which fuse together a discriminative approach (to get good initialisation and robustness) with a robust silhouette-free kinematic model fitting approach for precision.

A fast, approximate pose estimation system has previously been combined with a slow, more-accurate technique in order to provide basic parameters to athletics coaches and inform training in real-time [76]. This type of system may have utility in the applied field by allowing some of the primary, “top-level” biomechanical determinants (for example, step frequency and step length in gait) to be fed-back during normal training or rehabilitation situations. Importantly, the more complex and computationally expensive kinematic variables (such as 3D joint angles, which require modelling of the body) may still be acquired. However, the likelihood is that more time-consuming, offline processing will be necessary. This two-part approach could help address the apparent disconnect between sports science research and practice [114] as short participant preparation times and timely feedback from the system may increase the perceived (and actual) value of such studies to those operating in the applied field. Importantly, the more complex kinematic information could still be computed and communicated to applied practitioners across longer time frames, but equally these data can be used in research studies to continually progress our scientific understanding of human movement.

It should be noted that resolution (both spatial and temporal) will affect the accuracy of markerless systems in the same way as it does for marker-based systems. However, video-based automatic systems must also consider the fact that the size of the data captured will be considerably larger and thus, markerless systems may need to compromise accuracy to make a deployable, fast system feasible. Such a system requires large amounts of video data to be handled efficiently and effectively, which is likely to necessitate the purchase of an expensive (perhaps specially engineered) video-based system (e.g. machine vision).

## Conclusions

Vision-based motion analysis methods within sports and rehabilitation applications have evolved substantially over recent times and have allowed biomechanical research to contribute a vast amount of meaningful information to these fields. However, the most widespread kinematic data capture techniques (marker-based technologies and manual digitisation) are not without their drawbacks. Considerable developments in computer vision have sparked interest in markerless motion analysis and its possible wider applications. Although this potential is promising, it is not yet clear exactly what accuracy can be achieved and whether such systems can be effectively and routinely utilised in field-based (more externally valid) settings. Over the coming years, collaborative research between computer vision experts and biomechanists is required to further develop markerless techniques so they are able to meet the unique practical and accuracy requirements of motion analysis within sports and rehabilitation contexts.

## Abbreviations

2D: Two-dimensional
3D: Three-dimensional
DOF: Degrees of freedom
RMS: Root mean square
SMPL: Skinned Multi-Person Linear Model
